# Inducible Cre transgenic mouse strain for skeletal muscle-specific gene targeting

**DOI:** 10.1186/2044-5040-2-8

**Published:** 2012-05-07

**Authors:** John J McCarthy, Ratchakrit Srikuea, Tyler J Kirby, Charlotte A Peterson, Karyn A Esser

**Affiliations:** 1 Center for Muscle Biology, University of Kentucky, Lexington, KY, 40536, USA; 2 Department of Physiology, College of Medicine, University of Kentucky, Lexington, KY, 40536, USA; 3 Integrated Biomedical Sciences Program, College of Medicine, University of Kentucky, Lexington, KY, 40536, USA; 4 Department of Rehabilitation Sciences, College of Health Sciences, University of Kentucky, Lexington, KY, 40536, USA; 5 Department of Physiology, Faculty of Science, Mahidol University, Bangkok, 10400, Thailand; 6 Department of Physiology, College of Medicine, University of Kentucky, 800 Rose Street, Lexington, KY, 40536, USA

**Keywords:** Skeletal muscle-specific, Cre recombinase, Inducible

## Abstract

**Background:**

The use of the Cre/loxP system for gene targeting has been proven to be a powerful tool for understanding gene function. The purpose of this study was to create and characterize an inducible, skeletal muscle-specific Cre transgenic mouse strain.

**Methods:**

To achieve skeletal muscle-specific expression, the human α-skeletal actin promoter was used to drive expression of a chimeric Cre recombinase containing two mutated estrogen receptor ligand-binding domains.

**Results:**

Western blot analysis, PCR and β-galactosidase staining confirmed that Cre-mediated recombination was restricted to limb and craniofacial skeletal muscles only after tamoxifen administration.

**Conclusions:**

A transgenic mouse was created that allows inducible, gene targeting of floxed genes in adult skeletal muscle of different developmental origins. This new mouse will be of great utility to the skeletal muscle community.

## **Background**

The ability to manipulate the murine genome has proven to be instrumental in the understanding of gene function in vivo. In particular, the use of the Cre/loxP system has allowed investigators to circumvent the limitations of conventional gene targeting strategy by providing temporal and tissue-specific control over gene expression [1]. A number of different skeletal muscle-specific Cre mice have been used to alter gene expression during embryonic development and in the adult. In the most popular strains, the muscle creatine kinase (MCK), human α-skeletal actin (HSA), myogenic factor 5 (Myf5), myosin light chain 1/3 fast (MLC1/3f), myogenic differentiation 1 (Myod1), myogenin (Myog) or paired box gene 7 (Pax7) promoters have been used to drive expression [2-18]. A search of the CREATE consortium database (http://www.creline.org/) revealed that, of the dozen or so skeletal muscle fiber-specific Cre mice available, only two were inducible. One strain used a mutated estrogen receptor ligand-binding domain (CreMer) to control the timing of Cre-mediated recombination via tamoxifen activation [3]. The second inducible mouse strain employed a muscle-specific Tet-On system to control Cre expression following administration of the tetracycline analogue doxycycline [18].

To provide a readily available mouse strain to the skeletal muscle community, we generated our own skeletal muscle-specific, inducible Cre strain and characterized the effectiveness of recombination in both limb and craniofacial muscles. The design of our transgene was based on a previous Cre transgenic strain that achieved a high degree of skeletal muscle specificity using the HSA promoter [2]. We modified the previously described HSA-Cre transgene by substituting Cre with a MerCreMer (MCM) cDNA, thus making the system inducible by requiring tamoxifen binding to induce Cre-mediated recombination [2]. Characterization of the HSA-MCM mouse demonstrated skeletal muscle-specific expression of the MCM protein and that recombination only occurred following tamoxifen administration. Moreover, in addition to limb muscle, we observed recombination in craniofacial muscle, thus expanding the utility of this mouse strain for the study of gene function in skeletal muscles of different developmental origins.

## **Methods**

### **Cloning of the HSA-MerCreMer transgene**

The design of the transgene is based on a previous muscle-specific *Cre* transgene reported by Miniou and colleagues (1999); however, we replaced the Cre cDNA with an inducible form of *Cre* described by Verrou *et al.* (1999) [2,19]. The inducible *Cre* contained a mutated estrogen receptor (Mer) ligand-binding domain at both the N- and C-termini and was designated as MerCreMer (MCM) to be consistent with the cardiac-specific MCM strain [20]. To generate the transgene, the promoter and first exon (−2,000 to +239 relative to the transcription start site) of the HSA gene was amplified from human genomic DNA (Promega, Madison, WI, USA) and cloned into *Cla*I site of the SG5 expression vector (Agilent Technologies, Santa Clara, CA, USA) upstream of the β-globin intron II. The MCM cDNA was then amplified from the pANMerCreMer expression vector (a kind gift from Dr Reth) and cloned into the EcoRI site of the pSG5-HSA plasmid to generate the pSG5-HSA-MCM. The mutation (G525R) introduced into the estrogen receptor ligand-binding domain has been shown to abolish estradiol binding while retaining the ability to bind 4-hydroxytamoxifen [21]. The plasmid was then sequenced for verification. The HSA-MCM transgene was released from the plasmid by *Hin*dIII/*Nsi*I enzyme digestion, gel-purified using the QIAquick Gel Extraction Kit according to the manufacturer’s directions (Qiagen, Valencia, CA, USA) and then provided to the University of Kentucky Transgenic Mouse facility for microinjection.

### **Generation and screening of HSA-MCM transgenic lines**

**Figure 1:**
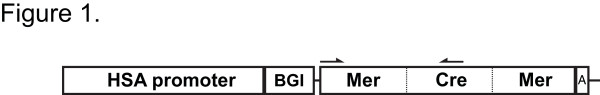
**A schematic of the HSA-MCM transgene**. The promoter and first exon (−2,000 to +239 relative to the transcription start site) of the human α-skeletal actin (HSA) gene drives expression of the MerCreMer (MCM) gene which harbors a mutated estrogen receptor (Mer) ligand-binding domain on each terminus of the *Cre* recombinase gene. The β-globin intron ΙΙ (BGI) and poly(A) tail were incorporated into the transgene to ensure proper splicing and transcript stability, respectively. The positions of the PCR primers used for genotyping are indicated by half-arrows.

All animal procedures were conducted in accordance with institutional guidelines for the care and use of laboratory animals as approved by the Institutional Animal Care and Use Committee of the University of Kentucky. The HSA-MCM transgene was introduced into F2 embryos derived from the mating of C57BL/6 X C3H (B6C3F1) parents. Production of mice was performed by the staff of the University of Kentucky Transgenic Mouse facility. Genomic DNA was isolated from tail biopsies of eight offspring using the DNeasy Blood & Tissue Kit (Qiagen) and screened for the presence of the HSA-MCM transgene by PCR using the following primers: forward, 5′-GCATGGTGGAGATCTTTGA-3′; reverse, 5′-GCTTCTGTCCG TTTGCCGGTCG-3′. The primers spanned the C-terminus MerCre junction (see Figure 1) and produced a 717-bp product. Five of the offspring were positive for the presence of the HSA-MCM transgene. To determine which of the HSA-MCM founder lines showed germline transmission and were capable of inducible, muscle-specific *Cre*-mediated recombination, each line was bred to a lacZ reporter mouse line (B6;129 S4-*Gt(ROSA)26Sor*^*tm1Sor*^/J, stock number 003474) purchased from The Jackson Laboratory (Bar Harbor, ME, USA) and originally described by Soriano [22]. A second reporter mouse strain, containing a *lacZ* with a nuclear localization signal, was also used to assess the ability of the HSA-MCM strain to drive inducible recombination and label adult skeletal muscle nuclei. This second reporter mouse was described by Yamamoto and colleagues and purchased from The Jackson Laboratory (FVB.Cg-*Gt(ROSA)26Sor*^*tm1(CAG-lacZ,-EGFP)Glh*^/J, stock number 012429) [23].

### **Western blot analysis**

To expand the potential utility of the HSA-MCM strain, we determined the expression of the MCM protein in skeletal muscles of different developmental origins. To generate total protein lysates for Western blot analysis, tissue samples were collected from limb muscles (Gstn, gastrocnemius; Pln, plantaris; Sol, soleus; EDL, extensor digitorum longus; Quad, quadriceps; TA, tibialis anterior), craniofacial muscles (EOM, extraocular muscle; Mastr, masseter; Tong, tongue), the heart (Hrt), samples containing smooth muscle (Stom, stomach; S. Int, small intestine) and nonmuscle tissue (Lung; Panc, pancreas; Liver; Brain; Fat; Spln, spleen; Kdny, kidney). Tissue samples (about 20 mg) were homogenized by using the Polytron PowerGen 125 (Fischer Scientific, Suwanee, GA, USA) in homogenization buffer (1% Nonidet P-40, 0.5% sodium deoxycholate, 0.1% SDS, 50 mM NaCl, 400 mM KCl, 25 mM β-glycerophosphate, 50 mM NaF, 5 mM benzamidine, 20 mM Tris·HCl (pH 7.6), 1 mM ethylenediaminetetraacetic acid, 1 mM sodium orthovanadate, 5 mM *N*-ethylmaleimide, 1 mM phenylmethylsulfonyl fluoride) supplemented with protease inhibitor cocktail (catalog no. P8340; Sigma-Aldrich, St Louis, MO, USA). The muscle homogenates were then centrifuged for 10 min at 10,000 *g* at 4°C, and the protein concentration of the supernatant was determined using the Bradford protein assay (Bio-Rad Laboratories, Hercules, CA, USA). Ten micrograms per sample were separated by SDS-PAGE (8% gel) and then transferred to nitrocellulose membrane (0.2 μm) (Bio-Rad Laboratories). The membrane was incubated in blocking buffer (5% nonfat dry milk in Tris-buffered saline (TBS) plus 0.1% Tween-20 (TBS-T)) for 1 hour at room temperature and then incubated in blocking buffer overnight at 4°C with a 1:3,000 dilution of the primary antibody. An antibody against the estrogen receptor-α (ERα) (MC-20; Santa Cruz Biotechnology, Santa Cruz, CA, USA) was used to detect the MCM protein and antibodies against γ-tubulin and glucose-6-phosphate dehydrogenase (T359 and A-9527, respectively; Sigma-Aldrich) were used to evaluate loading between samples. The ERα antibody was able to distinguish between the endogenous ERα (66 kDa) and MCM (112 kDa) proteins based on their significant difference in molecular weight as previously shown [19]. After the overnight incubation, the membrane was washed for 5 minutes four times in TBS-T and then incubated with a horseradish peroxidase-conjugated secondary antibody (2 ng/ml) for 45 minutes at room temperature in blocking buffer. Following this incubation, the membrane was washed again in TBS-T as described above, incubated for 5 minutes in chemiluminescence substrate (ECL Primer Western Blotting Detection Reagent; GE Healthcare, Piscataway, NJ, USA) and then visualized by exposure to X-ray film.

### **β-galactosidase assay**

Tissue was excised and mounted on an aluminum-covered cork block, covered in O.C.T. compound, frozen in liquid nitrogen-cooled isopentane and then stored at −80°C until sectioning. Tissue sections (10 μm) were air-dried for 30 minutes, rehydrated in PBS for 10 minutes, fixed in 0.2% glutaraldehyde for 7 minutes at room temperature and then washed briefly three times in PBS. Fixed sections were then incubated overnight in 5-bromo-4-chloro-3-indolyl-β-D-galactopyranoside (X-gal) working solution at 37°C in a humidified chamber. The X-gal working solution contained 5 mM potassium hexacyanoferrate(III), 5 mM potassium hexacyanoferrate(II) trihydrate, 2 mM MgCl_2_ and 1 mg/ml of X-gal. Following the overnight incubation, sections were washed three times for 5 minutes per wash in PBS, dehydrated in 95% ethanol for 1 minute twice, 100% ethanol for 1 minute twice, cleared for 1 minute in xylene and then mounted on a coverslip using Permount mounting media. For nuclear localized β-galactosidase detection, transcardial perfusion was performed using ice-cold PBS containing 10 U of heparin followed by freshly prepared, ice-cold 4% paraformaldehyde. The Gstn muscle was dissected out and fixed for an additional 60 minutes in 4% paraformaldehyde, which was followed by a series of rinses in PBS. Tissue was cryoprotected by then being placed in a 15% (wt/vol) sucrose solution until equilibration, followed by immersion in a 30% sucrose solution until equilibration, each performed at 4°C. Tissue was then transferred to a 1:1 (vol/vol) mixture of 30% sucrose and O.C.T. compound (Tissue-Tek; Sakura Finetek USA, Inc, Torrance, CA,) for 30 minutes and then embedded in O.C.T. compound and frozen in an ethanol, dry-ice solution. Tissue sections were viewed using a Nikon E600 microscope (Nikon Inc, Melville, NY, USA), and images were captured with a SPOT RT digital camera (Diagnostic Instruments, Inc, Sterling Heights, MI, USA) and a PowerMac G4 computer (Apple Computer Inc, Cupertino, CA, USA) equipped with SPOT RT software version 4.0 (Diagnostic Instruments, Inc).

### **PCR analysis of cre-mediated recombination**

PCR was performed to assess recombination following tamoxifen administration. The PCR conditions and primer sequences used were as described by Takehashi *et al.*[24].

## **Results and discussion**

A schematic of the HSA-MCM transgene is presented in Figure 1. We used the HSA promoter (−2,000 to +239) to drive skeletal muscle-specific expression of the Cre cDNA as others have done [2]. Additionally, the β-globin intron II was incorporated into the transgene to ensure proper splicing [2,25]. To make the system inducible, we replaced the Cre cDNA with a modified Cre that contained a Mer ligand-binding domain at both N- and C-termini, thereby creating the MCM chimeric protein [19,21]. We decided to use the MCM protein based on the finding that it has been shown to be more tightly regulated (that is, less recombinant in the absence of tamoxifen) than the single CreMer fusion protein, with no loss in recombination efficiency [19] .

**Figure 2:**
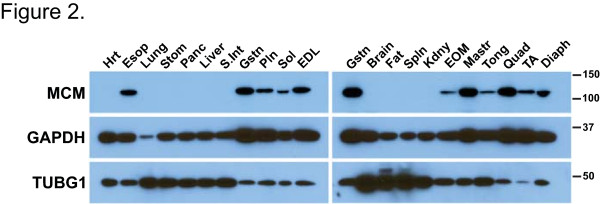
**Skeletal muscle-specific expression of MerCreMer (MCM) protein.** Western blot analysis of different muscle and nonmuscle tissues from the human α-skeletal actin (HSA)-MCM transgenic strain detected the MCM protein (112 kDa) only in skeletal muscle (Esop, esophagus; Gstn, gastrocnemius; Pln, plantaris; Sol, soleus; EDL, extensor digitorum longus; EOM, extraocular muscle; Mastr, masseter; Tong, tongue; Quad, quadriceps; TA, tibialis anterior; Diaph, diaphragm) and not in the heart (Hrt), samples containing smooth muscle (Stom, stomach; S. Int, small intestine) or nonmuscle tissue (Lung; Panc, pancreas; Liver; Brain; Fat; Spln, spleen; Kdny, kidney). Both glyceraldehyde 3-phosphate dehydrogenase (GAPDH) and tubulin, γ1 (TUBG1) were used as loading controls.

To confirm the ability of the HSA promoter to drive skeletal muscle-specific expression of the MCM protein, Western blot analysis was performed using protein extracts derived from a broad range of muscles as well as from nonmuscle tissues. As shown in Figure 2, the MCM protein was detected only in skeletal muscle samples and not in the heart (Hrt) or in samples containing smooth muscle (Stom, stomach; S. Int, small intestine). Importantly, in addition to limb musculature (Gstn, gastrocnemius; Pln, plantaris; Sol, soleus; EDL, extensor digitorum longus; Quad, quadriceps; TA, tibialis anterior), the MCM protein was detected in other skeletal muscles, including the diaphragm (Diaph), extraocular muscle (EOM), masseter (Mastr), tongue (Tong) and esophagus (Esop). The expression of MCM protein in craniofacial muscles (EOM, Mastr and Tong) expands the utility of the HSA-MCM mouse for investigators interested in studying gene function in craniofacial muscles.

**Figure 3:**
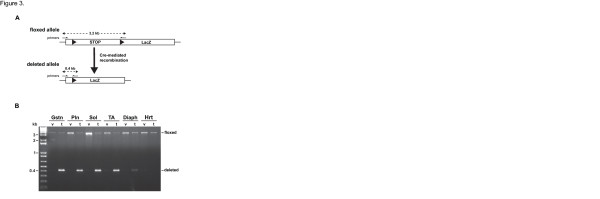
**PCR analysis of inducible, skeletal muscle-specific, Cre-mediated recombination. (A)** Schematic of the *lacZ* reporter gene showing the floxed (flanking loxP sites, solid arrowheads) STOP cassette prevents expression of the downstream *lacZ* cDNA. Following tamoxifen administration, a Cre-mediated recombination event resulted in deletion of the STOP cassette, thereby allowing expression of the *lacZ* cDNA. **(B)** Qualitative PCR analysis of genomic DNA shows Cre-mediated recombination (deleted, 0.4-kb band) occurred only in skeletal muscle samples (Gstn, gastrocnemius; Pln, plantaris; Sol, soleus; TA, tibialis anterior; Diaph, diaphragm) and not in the heart (Hrt) following tamoxifen (t) administration, with no recombination (floxed, 3.2-kb band) in vehicle-treated (v) samples. The positions of the PCR primers are indicated by half-arrows.

Having established the skeletal muscle-specific expression of the MCM protein, we next wanted to determine the effectiveness of the system to mediate recombination in response to tamoxifen administration. To provide a readout of recombination, the HSA-MCM mouse was bred to an inducible *lacZ* reporter strain [22]. A schematic of the *lacZ* reporter gene is shown in Figure 3A. Expression of the *lacZ* gene is prevented by the upstream presence of a STOP cassette that is flanked by loxP (floxed) sites. Upon exposure to tamoxifen, Cre promotes a recombination event that leads to excision of the STOP cassette and expression of *lacZ* cDNA. PCR analysis was employed to detect this recombination event using genomic DNA isolated from different skeletal muscles as a template. As shown in Figure 3B, recombination was detected only in skeletal muscle samples following tamoxifen administration, consistent with the expression of the MCM protein. These results demonstrate the MCM protein is capable of effective recombination that is tightly regulated, as no recombination event was detected in vehicle-treated samples.

**Figure 4:**
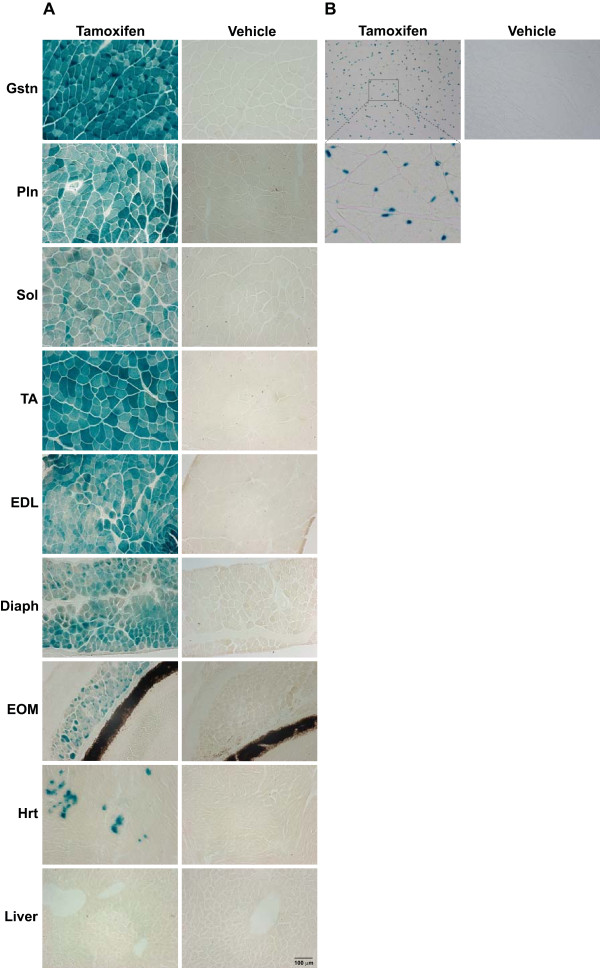
**Skeletal muscle-specific recombination as assessed by β-galactosidase activity.** The HSA-MCM strain was bred to a *lacZ* reporter mouse to visually assess tamoxifen-induced recombination. **(A)** consistent with the Western blot analysis and PCR results, strong β-galactosidase expression (blue precipitate) was observed only in skeletal muscle (Gstn, gastrocnemius; Pln, plantaris; Sol, soleus; TA, tibialis anterior; EDL, extensor digitorum longus; Diaph, diaphragm; EOM, extraocular muscle) and not in the heart (Hrt) or liver following tamoxifen administration. **(B)** When we used a second lacZ reporter mouse (that contained a nuclear localization signal), β-galactosidase-positive, “blue” myonuclei were observed in Gstn skeletal muscle after tamoxifen treatment, but not in vehicle-treated Gstn. Inset shows enlarged image of labeled nuclei that reside within the muscle fiber.

To localize Cre-mediated recombination at the cellular level, we performed histology to detect *LacZ* expression by assaying β-galactosidase activity following addition of the X-gal substrate. Consistent with the Western blot and PCR analyses, we observed X-gal staining in all skeletal muscle fibers of tamoxifen-treated mice (see Figure 4A).Importantly, no X-gal staining was observed in vehicle-treated samples, confirming that the MCM-mediated recombination requires tamoxifen treatment. We did, however, detect low-level X-gal staining in the heart, and, though this finding was somewhat surprising because of the lack of detection of the MCM protein in the heart, it is consistent with the findings of Collins *et al.*[26]. Using a quantitative Northern blot method, Collins and colleagues found a low level of α-skeletal actin mRNA in the adult mouse heart [26]. Thus, the minor amount of X-gal staining in the heart likely reflects the function of the HSA promoter rather than a transgenic artifact or leakiness of the MCM system.

Although all skeletal muscles were positive for X-gal staining, variability in the intensity of staining among different skeletal muscle samples was apparent. It is important to note that there does not appear to be a linear relationship between X-gal staining intensity and the level of recombination, as our PCR results show similar levels of recombination in the soleus and gastrocnemius muscles; yet the staining intensities between these two muscles are different. Moreover, there does not appear to be any relationship between X-gal staining intensity and either type I or type II fiber composition, consistent with what has been reported for other HSA-Cre strains [2,3]. For example, the Gstn and Pln muscles are composed of > 90% type II fibers, but X-gal staining intensity is much stronger in Gstn than Pln muscle fibers (see Figure 4) [27]. The variability in the intensity of X-gal staining among the different muscle samples likely reflects differences in the activity of the Rosa26 promoter and/or stability of the *E. coli* β-galactosidase protein across the different muscles.

The HSA-MCM mouse is also useful for driving reporter genes with different subcellular localization in muscle fibers, as demonstrated in Figure 4B. The HSA-MCM mouse was bred to a lacZ reporter mouse that contained a nuclear localization signal. X-gal staining on gastrocnemius (Gstn) muscle sections showed “blue” myonuclei were detectable following tamoxifen treatment (Figure 4B). Furthermore, there are no labeled myonuclei in the muscle from vehicle-treated mice, indicating that the system is tightly regulated at this age (12 to 14 weeks of age). To provide an additional measure of recombination efficiency, we counted the number of X-gal-positive nuclei associated with approximately 900 fibers from the Gstn muscle. On the basis of the results of this analysis, we determined that there are approximately 0.91 myonuclei per fiber, a value comparable to the 0.88 myonuclei per fiber we recently reported [28]. Although these results demonstrate that the HSA-MCM strain can achieve a high level of recombination efficiency, they should be interpreted with caution as there is evidence suggesting that the β-galactosidase protein can translocate to “unlabeled” nuclei [29].

## **Conclusions**

Collectively, the results of our study provide convincing evidence that the HSA-MCM strain allows robust, tightly controlled, Cre-mediated recombination specifically within adult skeletal muscle in an inducible manner. Furthermore, the ability of the HSA promoter to drive MCM expression in craniofacial muscles, in addition to limb musculature, greatly expands the usefulness of the HSA-MCM mouse strain to the skeletal muscle community. The HSA-MCM mouse will be freely available upon request.

## **Abbreviations**

bp: Base pair; HSA: Human skeletal muscle actin; kb: Kilobase; kDa: Kilodalton; Mer: Mutated estrogen receptor; MCM: MerCreMer; PBS: Phosphate-buffered saline; PCR: Polymerase chain reaction; SDS: Sodium dodecyl sulfate.

## **Competing interests**

The authors have no financial or nonfinancial competing interests to declare.

## **Authors’ contributions**

JJM cloned the transgene, characterized the HSA-MCM mouse and wrote the manuscript. RS and TK assisted in the characterization of the mouse. KAE and CAP developed the experimental design and edited the manuscript. All authors read and approved the final manuscript.
