# Correction: Inducible Cre transgenic mouse strain for skeletal muscle-specific gene targeting

**DOI:** 10.1186/2044-5040-2-22

**Published:** 2012-10-30

**Authors:** John J McCarthy, Ratchakrit Srikuea, Tyler J Kirby, Charlotte A Peterson, Karyn A Esser

**Affiliations:** 1 Center for Muscle Biology, University of Kentucky, Lexington, KY 40536, USA; 2 Department of Physiology, College of Medicine, University of Kentucky, Lexington, KY 40536, USA; 3 Integrated Biomedical Sciences Program, College of Medicine, University of Kentucky, Lexington, KY, 40536, USA; 4 Department of Rehabilitation Sciences, College of Health Sciences, University of Kentucky, Lexington, KY, 40536, USA; 5 Department of Physiology, Faculty of Science, Mahidol University, Bangkok, 10400, Thailand

## 

*Skeletal Muscle* 2012, **2**:8 http://dx.doi.org/10.1186/2044-5040-2-8.

The electronic version of this article is the complete one and can be found online at: http://www.skeletalmusclejournal.com/content/2/1/8.

Correction

Upon publication of this work [1], it was brought to our attention that the reverse primer sequence for genotyping of HSA-MCM pups was incorrect. The correct reverse primer sequence is 5^′^-CGACCGGCAAACGGACAGAAGC −3^′^.
